# Awareness of the General Population Toward Constipation and Its Complications in the Western Region, Saudi Arabia

**DOI:** 10.7759/cureus.36022

**Published:** 2023-03-11

**Authors:** Mohannad Hemdi, Mayar Y Alkarmo, Raneem A Alahmadi, Rawaan S Almajnoni, Jana K Alharbi, Amal M Alfahmi, Hatim A Almaghrabi

**Affiliations:** 1 Department of General Surgery, Umm Al-Qura University, Makkah, SAU; 2 Department of Medicine and Surgery, Umm Al-Qura University, Makkah, SAU

**Keywords:** saudi arabia, western region, adult, awareness, constipation

## Abstract

Background and aim

Constipation can be defined as unsatisfying defecation characterized by difficult stool passage, infrequent stools, or both. Complications include hemorrhoids, anal fissures, and prolapse of pelvic organs. Unfortunately, there needs to be more data available regarding awareness on this subject. Thus, in this study, we aim to measure the level of understanding of constipation and its complications in the western region of Saudi Arabia.

Methods

In this cross-sectional study, a valid and reliable questionnaire was used in data collection. In addition, SPSS (IBM Corp., Armonk, NY) was used for the analysis of collected data.

Results

The present study included a total of 778 participants from the general public, 75.6% of whom were female and 24.4% of whom were male. We found that 70% of participants had an overall good awareness of constipation, with significant variation in levels of awareness among different groups; levels of awareness increased with age (P-value < 0.001), higher education also positively affected the level of understanding (P-value = 0.04), and participants who reported personal experience had higher levels of awareness than participants who did not have personal experience (P-value​​​​​​​ = 0.002). There was no significant association between the level of awareness and gender or city of residence.

Conclusion

Much of our population was well aware of constipation and its complications, with significant variation in awareness among different age groups, educational levels, and personal experiences.

## Introduction

The American College of Gastroenterology defines constipation as unsatisfying defecation characterized by difficulty passing stools, infrequent stools, or both [1]. It is usually described as severe straining, a sensation of incomplete evacuation, hard stools, fewer stools per week, and prolonged or failed attempts at defecation. It can be chronic or acute, and in terms of etiology, it can be either primary or secondary [2-4]. The physiological risk factors for constipation include advanced age, obesity, female gender, anatomical abnormalities, pregnancy, and constipation secondary to illness or diseases [5]. Among other complications, the most common complication of constipation is hemorrhoids. Hemorrhoids are swollen veins in the rectum and anus that can cause pain, bleeding, and itching. Other complications include anal fissures and prolapse of pelvic organs such as the rectum, urinary bladder, uterus, and vagina. The Rome IV diagnostic criteria are used in adults with no suspected or identified causes of secondary constipation [6]. Furthermore, advanced investigations include an anorectal function test to evaluate defecatory abnormalities. In contrast, colonic transit studies are used to distinguish between normal transit constipation and slow transit constipation [7-9].

A cross-sectional study of 1,855 participants in Riyadh to evaluate the general population's awareness of constipation reported that 75% of participants were well aware of constipation and its complications. With a significant difference between the level of awareness among different age groups. Additionally, there was a strong correlation between marital status and the level of awareness [8]. Another study conducted in 2019 with 543 participants from the central region of Saudi Arabia reported that the prevalence of constipation among the sampled participants was only 4.4%, whereas 95.6% indicated they were not suffering from constipation. However, constipation was more prevalent among females (79.2%) than males (20.8%). Moreover, constipation is more severe among those between 20 and 35 years old, while it reaches 0% among older people (over 51 years). The results also show that constipation is more prevalent among people who consume fiber-rich foods once a week, are constantly stressed, dehydrated, and are non-smokers [9].

However, to the best of our knowledge, there have yet to be any previous studies in Makkah to assess the awareness of constipation and its complications in our society. Therefore, it is essential to properly research this topic due to the condition’s prevalence and how significantly it can interfere with an individual's life and well-being. Therefore, we aimed to assess the general public’s knowledge of constipation and its associated complications.

## Materials and methods

Study design and setting

This is a cross-sectional study conducted using an online validated questionnaire, which was prepared for distribution using Google Forms (Google, Mountain View, CA). The study was conducted among the general population of the western region of Saudi Arabia from October 2022 to November 2022.

Ethical consideration

We distributed our survey after we obtained ethical approval on October 10, 2022, from the Biomedical Research Ethics Committee of Umm Al-Qura University, Makkah, Saudi Arabia (approval number: HAPO-02-K-012-2022-09-1210).

The subjects were informed of their rights to refuse participation and to end their involvement at any time. In addition, the study objectives, the methods that would be utilized to collect the data, and the assurance of the safety of participants were all explained to the subjects.

In this study, confidentiality and anonymity were maintained by not disclosing the participant’s name on the questionnaire and research reports, keeping the collected data confidential, and not revealing the subjects’ identities.

Eligibility criteria

Since our study targeted the general population of the western region in Saudi Arabia, we included all the males and females of the defined population aged more than 18 years. However, healthcare workers and people who refused to participate in the survey were excluded from the study.

Sample size

After determining our study population, we calculated the sample size using OpenEpi version 3.0. Accordingly, the sample size required for this study was 385. However, we succeeded in involving 778 participants in our research.

Study tool

This study used English and Arabic versions of a previously published, validated questionnaire modified to suit our targeted population. The English version was used for statistics and the Arabic version for data collection. The survey is divided into two sections, including demographic questions in the first section, while the second assesses awareness of constipation and its complications. A consent form was obtained from the participants at the beginning of the study questionnaire.

Statistical analysis

A standard scoring method was used to assess participants’ knowledge; one point was given for correct answers, and 0 for incorrect and “I do not know’’ responses. After data collection, participants who correctly answered 60% or more of the questions were considered to have good knowledge about constipation.

We entered the data on Microsoft Excel (Microsoft Corporation, Redmond, WA) spreadsheets. The data were transferred into spreadsheets of Statistical Package for the Social Sciences (SPSS, version 26, IBM Corp., Armonk, NY). Frequency was calculated for categorical variables and mean ± standard deviation for continuous variables. For comparing categorical variables, the chi-square test was used. Univariate analysis was done to find the association between the level of awareness and age, gender, city of residence, education, and personal experience. A P-value of <0.05 was considered significant.

## Results

A total of 778 adults from the western region of Saudi Arabia were included in this study. Of the participants, 75.6% were female, and 24.4% were male. Participants of different age groups and cities of the western region were included. In addition, 74.4% of the participants held bachelor’s degrees, which makes most of the study population well-educated. Additional socio-demographic data are demonstrated in Table 1.

**Table 1:** Socio-demographic characteristics of study participants

Characteristic		Frequency	Percentage
Gender	Female	588	75.6%
	Male	190	24.4%
Age	≤20	150	19.3%
	21-30	323	41.5%
	31-40	146	18.8%
	41-50	110	14.1%
	51-60	39	5.0%
	>60	10	1.3%
City of residence	Makkah	165	21.2%
	Medina	292	37.5%
	Jeddah	120	15.4%
	Taif	116	14.9%
	Yanbu	85	10.9%
Level of education	No formal education	8	1.0%
	Elementary school	5	0.6%
	Intermediate school	25	3.2%
	High school	161	20.7%
	Bachelor	579	74.4%

**Figure 1:**
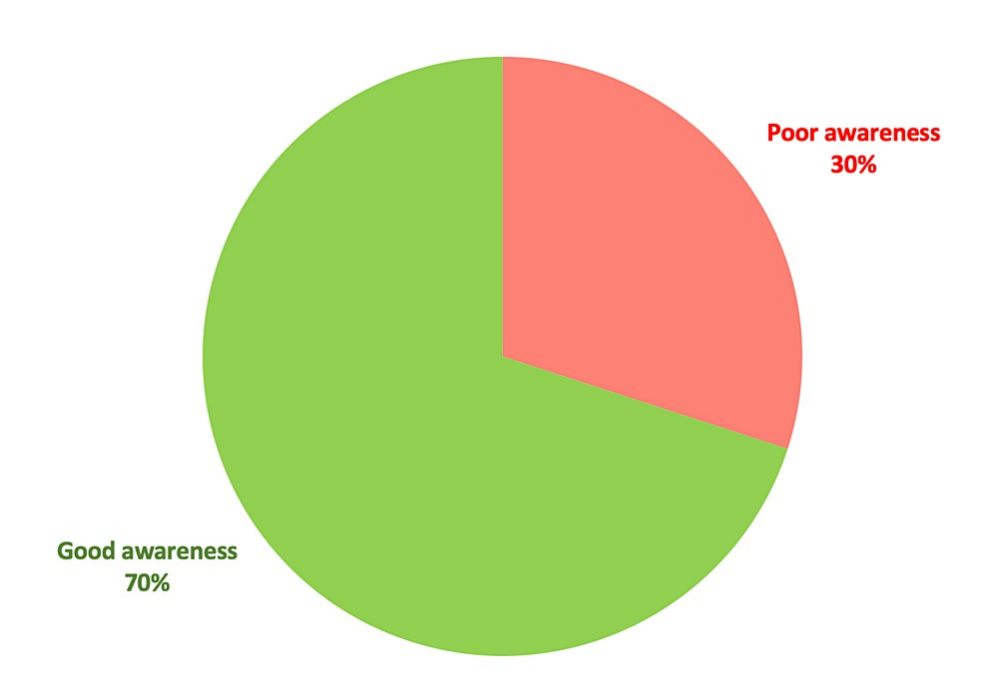
Awareness of constipation

A detailed description of the participants’ answers is shown in Table 2. Analysis of responses showed that 70% of participants were aware of constipation and its complications, as shown in Figure 1.

**Table 2:** Participants’ awareness of constipation and its complications

Statement	Agree	Disagree	I don’t know
1. Constipation means that a person has three or fewer bowel movements in a week	490 (63.0%)	173 (22.2%)	115 (14.8%)
2. Constipation is a common condition in Saudi Arabia	501 (64.4%)	50 (6.4%)	227 (29.2%)
3. Lack of exercise is a risk factor in the development of constipation	549 (70.6%)	79 (10.2%)	150 (19.3%)
4. Advanced age increases the likelihood of developing problems associated with bowel movements	600 (77.1%)	63 (8.1%)	115 (14.8%)
5. inadequate dietary fiber is a predisposing factor in the development of constipation	672 (86.4%)	27 (3.5%)	79 (10.2%)
6. Constipation could be due to idiopathic/unknown causes	462 (59.4%)	134 (17.2%)	182 (23.4%)
7. Causes of constipation vary between simple dietary causes to severe malignancy causes	513 (65.9%)	54 (6.9%)	211 (27.1%)
8. Constipation is a significant risk factor for developing anorectal conditions like (hemorrhoids, hernias, and anal fissures)	670 (86.1%)	23 (3.0%)	85 (10.9%)
9. Excruciating abdominal pain associated with complete constipation requires emergent medical/surgical care	448 (57.6%)	123 (15.8%)	207 (26.6%)
10. Adequate water intake is critical in preventing the development of constipation	708 (91.0%)	24 (3.1%)	46 (5.9%)
11. Regular visit to primary care is crucial in the management of constipation and its related complications	606 (77.9%)	68 (8.7%)	104 (13.4%)
12. The use of over-the-counter constipation treatment is effective in the treatment of constipation	246 (31.6%)	147 (18.9%)	385 (49.5%)
13. The cost of treatment of constipation influences the decision to seek medical services	307 (39.5%)	145 (18.6%)	326 (41.9%)
Statement	Yes	No	I don’t know
14. Do you currently feel constipated, or have you had constipation in the past?	527 (67.7%)	232 (29.8%)	19 (2.4%)
15. Do you think constipation will affect a person’s quality of life?	595 (76.5%)	103 (13.2%)	80 (10.3%)

Table 3 shows the association between the level of awareness and gender, age, city of residence, level of education, and personal experience. Our results suggest an increased awareness with age (P-value < 0.001), with the group aged 41-50 years having the best understanding. The level of education also played a role in the level of awareness (P-value = 0.04). Participants who reported personal experience of constipation had the best understanding of constipation, followed by participants who reported a family history of constipation (P-value = 0.002). There was no significant association between the level of awareness with gender or city of residence.

**Table 3:** The relationship between the socio-demographic characteristics of participants and their level of awareness

Characteristic	Level of awareness				P-value
	Good awareness		Poor awareness		
	Frequency	Percentage	Frequency	Percentage	
Gender					
Male	125	65.8%	65	34.2%	0.181
Female	417	70.9%	171	29.1%	
Age					
≤20	78	52.0%	72	48.0%	0.00003
21-30	238	73.7%	85	26.3%	
31-40	110	75.3%	36	24.7%	
41-50	83	75.5%	27	24.5%	
51-60	26	66.7%	13	33.3%	
>60	7	70.0%	3	30.0%	
City of residence					
Makkah	111	67.3%	54	32.7%	0.455
Medina	207	70.9%	85	29.1%	
Jeddah	90	75.0%	30	25.0%	
Taif	75	64.7%	41	35.3%	
Yanbu	59	69.4%	26	30.6%	
Level of education					
No formal education	5	62.5%	3	37.5%	0.04
Elementary school	4	80.0%	1	20.0%	
Intermediate school	14	56.0%	11	44.0%	
High school	99	61.5%	62	38.5%	
Bachelor	420	72.5%	159	27.5%	
Personal experience					
Positive personal history	392	72.7%	147	27.3%	0.002
Positive family history	111	66.9%	55	33.1%	
Adverse personal or family history	39	53.4%	34	46.6%	

## Discussion

Although constipation is a common complaint that can be easily avoidable, it has numerous possible etiologies, including medical and surgical pathologies. This vast range of underlying pathologies makes it critical to study the issue. Studies have covered the awareness of constipation and its complications [8]. However, a study has yet to be conducted to assess the understanding of constipation and its complications among the western Saudi Arabian population.

Our present study included 778 adult participants of different age groups, cities of residence, and educational levels. We found that 70% of participants understood constipation and its complications well. Factors influencing their level of understanding included age, academic status, and having a personal or family experience with constipation.

The effect of these influencing factors should be accounted for in directing health promotion campaigns. Our results indicate that the young population (≤20 years old) needs the most attention regarding educational campaigns as they had the lowest levels of awareness; other groups needing educational campaigns include lower academic levels.

Most participants agreed with statements about the importance of suitable dietary fibers and water intake for preventing constipation and correctly identifying anorectal conditions like hemorrhoids, hernias, and anal fissures as possible complications for chronic constipation.

Our results are similar to previous studies in the region. For example, a study conducted in Riyadh in 2020 with 1855 participants showed that 75% of the respondents had a good awareness of constipation and its complications, with 86.4% of the study participants agreeing that insufficient dietary fiber intake is a risk factor for constipation, and 91% agreed that sufficient water intake is critical to prevent constipation [8].

This information is essential for the prevention of constipation, as findings reveal that constipation is more prevalent among those who do not have proper fiber and water intake [9]. In addition, another study conducted in Makkah reported that a significant association was found between the prevalence of constipation and dietary fiber consumption [5].

In the Saudi population, different studies have been performed, including the general population, to evaluate the prevalence of constipation, showing a percentage of 4.4% in the central region. Another study revealed that the prevalence of constipation is 22% in the Makkah region [5]. Our data reported that 67.7% of participants currently feel or have had constipation in the past.

Although the current study is the first to investigate the level of awareness regarding constipation in the western Saudi Arabian population, and despite the large sample size, limitations of this study include few responders above the age of 50 years, few uneducated participants, as well as fewer male participants in comparison to female participants.

## Conclusions

The current study found that much of the western Saudi Arabian population was aware of constipation and its complications, with significant variation in awareness among different age groups, educational levels, and personal experiences. Therefore, educational campaigns specifically targeting less aware populations are advised.

## References

[1] Brandt LJ, Prather CM, Quigley EM, Schiller LR, Schoenfeld P, Talley NJ (2005). Systematic review on the management of chronic constipation in North America. Am J Gastroenterol.

[2] Herz MJ, Kahan E, Zalevski S, Aframian R, Kuznitz D, Reichman S (1996). Constipation: a different entity for patients and doctors. Fam Pract.

[3] Rao SS, Tuteja AK, Vellema T, Kempf J, Stessman M (2004). Dyssynergic defecation: demographics, symptoms, stool patterns, and quality of life. J Clin Gastroenterol.

[4] Rao SS, Rattanakovit K, Patcharatrakul T (2016). Diagnosis and management of chronic constipation in adults. Nat Rev Gastroenterol Hepatol.

[5] Ali MH, Almuqati BS, Alhasnani HH, Alfahmi TR, Mandili AK, Shatla MM (2021). The prevalence and risk factors of constipation among the general population in Makkah, Saudi Arabia. IJMDC.

[6] Jamshed N, Lee ZE, Olden KW (2011). Diagnostic approach to chronic constipation in adults. Am Fam Physician.

[7] Bharucha AE, Dorn SD, Lembo A, Pressman A (2013). American Gastroenterological Association medical position statement on constipation. Gastroenterology.

[8] Ahmed SAS, Alshahrani AS, Alhazzaa AS, Alslaihem MF, Benragosh NJ, Alswayah MA (2020). Awareness of adult population toward constipation and its complications in Riyadh, Saudi Arabia. IJMDC.

[9] Alhassan M, Alhassan A, Alfarhood A, Alotaibi K, Alrashidy N, Alshalhoub K, Almeshal M (2019). Prevalence of constipation among central region population, Riyadh and Qassim provinces, Saudi Arabia, 2018-2019. J Family Med Prim Care.

